# Glutamine rapidly induces the expression of key transcription factor genes involved in nitrogen and stress responses in rice roots

**DOI:** 10.1186/s12864-015-1892-7

**Published:** 2015-09-25

**Authors:** Chia-Cheng Kan, Tsui-Yun Chung, Yan-An Juo, Ming-Hsiun Hsieh

**Affiliations:** Institute of Plant and Microbial Biology, Academia Sinica, Taipei, 11529 Taiwan

**Keywords:** Amino acid, Gene expression, Glutamine, Rice, Signal transduction, Transcription factor

## Abstract

**Background:**

Glutamine is a major amino donor for the synthesis of amino acids, nucleotides, and other nitrogen-containing compounds in all organisms. In addition to its role in nutrition and metabolism, glutamine can also function as a signaling molecule in bacteria, yeast, and humans. By contrast, the functions of glutamine in nutrition and as a signaling molecule remain unclear in plants.

**Results:**

We demonstrated that glutamine could effectively support the growth of rice seedlings. In glutamine-treated rice roots, the glutamine contents increased dramatically, whereas levels of glutamate remained relatively constant. Transcriptome analysis of rice roots revealed that glutamine induced the expression of at least 35 genes involved in metabolism, transport, signal transduction, and stress responses within 30 min. Interestingly, 10 of the 35 early glutamine responsive genes encode putative transcription factors, including two *LBD37*-*like* genes that are involved in the regulation of nitrogen metabolism. Glutamine also rapidly induced the expression of the *DREB1A*, *IRO2*, and *NAC5* transcription factor genes, which are involved in the regulation of stress responses.

**Conclusions:**

In addition to its role as a metabolic fuel, glutamine may also function as a signaling molecule to regulate gene expression in plants. The rapid induction of transcription factor genes suggests that glutamine may efficiently amplify its signal and interact with the other signal transduction pathways to regulate plant growth and stress responses. Thus, glutamine is a functional amino acid that plays important roles in plant nutrition and signal transduction.

**Electronic supplementary material:**

The online version of this article (doi:10.1186/s12864-015-1892-7) contains supplementary material, which is available to authorized users.

## Background

Most plants use inorganic nitrogen nitrate and ammonium as their primary nitrogen source [1]. Nitrate taken up from the soil is reduced to nitrite by nitrate reductase. Nitrite is very toxic and in the plant cell nitrite is quickly reduced to ammonium by nitrite reductase. Ammonium derived from nitrate or directly absorbed from the soil can be assimilated into glutamine and glutamate via the glutamine synthetase (GS)/glutamine-oxoglutarate aminotransferase (GOGAT) cycle [2]. In the GS/GOGAT cycle, GS catalyzes the synthesis of glutamine from ammonium and glutamate and GOGAT catalyzes the transfer of the amide group of glutamine to oxoglutarate to synthesize glutamate. The GS/GOGAT cycle is the major pathway for the primary nitrogen assimilation in plants [3]. In addition to primary nitrogen assimilation, GS and GOGAT also participate in the remobilization of nitrogen-containing compounds and the assimilation of large amounts of ammonium generated by photorespiration in C3 plants [4].

Plants can also take up organic nitrogen such as amino acids and peptides from the soil, although in natural environments, these organic compounds usually occur in lower amounts than nitrate and ammonium [5]. Glutamine is the first organic nitrogen compound derived from the assimilation of ammonium. Regardless of its origin, glutamine is one of the most abundant free amino acids in plants. In addition to protein and nucleotide biosynthesis, glutamine is a major amino donor for synthesis of amino acids and other nitrogen-containing compounds in plants [6].

Besides its role in nutrition and metabolism, glutamine also functions as a signaling molecule in the other organisms, including bacteria, yeast, and humans. In bacteria, the signaling role of glutamine is mainly related to metabolic regulation [7]. In enteric bacteria, glutamine synthetase (GlnA) is the primary enzyme involved in ammonium assimilation, which is tightly regulated at the transcriptional and posttranscriptional levels by the intracellular nitrogen status. The PII signal transduction protein GlnB, although not a direct sensor of glutamine, senses oxoglutarate and is tightly coupled to glutamine signaling [8]. The PII-modifying enzyme GlnD functions as the primary sensor of glutamine and uridylylates and deuridylylates PII in response to glutamine and oxoglutarate [9]. The PII-GlnD system has been conserved as the central control unit in nitrogen metabolism and glutamine signaling in bacteria [7].

PII homologs also exist in plants [10]. However, there is no evidence to show that plant PII-like proteins are modified by uridylylation, and GlnD homologs have yet to be identified in plants. Bacterial GlnD proteins contain two C-terminal ACT domains (named after aspartate kinase, chorismate mutase and TyrA) that are involved in sensing glutamine (9). Arabidopsis ACR proteins contain ACT domain repeats that have sequence similarity to the C-terminal region of bacterial GlnD, but the functions of these proteins are still unknown [11, 12]. It has been shown that the PII signal transduction protein is an ATPase and its activity is regulated by oxoglutarate [13]. A more recent study indicated that PII functions in glutamine sensing in many plants [14]. The plant-specific C-terminal extension of PII forms a low-affinity glutamine-binding site that is involved in the regulation of N-acetyl-glutamate kinase (NAGK) activity leading to the formation of arginine and polyamine in the chloroplast [14]. If plants have adapted the conserved PII-GlnD system in glutamine metabolism and signaling, it must have evolved into a form that significantly differs from that of bacteria [15].

In humans, glutamine is the most abundant free amino acid, present at estimated concentrations of 2–20 mM in different cells or tissues [16]. In addition to serving as a metabolic fuel and protein precursor, glutamine also regulates the expression of genes related to metabolism, cell defense, and signal transduction in humans [16, 17]. The molecular mechanisms involved in the control of gene expression by glutamine are still unclear. Nonetheless, increasing evidence suggests that glutamine may enhance many cell functions via the activation of various transcription factors in mammals and activation of these transcription factors may amplify glutamine signaling [16, 17]. The target of rapamycin complex 1 (TORC1) is an evolutionarily conserved master regulator of protein translation and cell growth [18]. The yeast TORC1 signaling pathway responds to intracellular levels of glutamine [19]. Glutamine also activates the mammalian TORC1 (mTORC1) to coordinate cell growth and proliferation [20]. These studies highlight the importance of glutamine as a signaling molecule in humans and yeast.

The regulatory and signaling functions of glutamine in plants remain to be explored and most studies on glutamine in plants have focused on its role as the primary amino acid derived from the assimilation of inorganic nitrogen [21, 22]. For instance, glutamine affects the uptake of nitrate and ammonium, and the activity of nitrate reductase [23–28]. In plants, glutamine also regulates the expression of nitrate reductase, nitrate transporter, and ammonium transporter genes [29–32]. Moreover, glutamine affects the expression of *NADH*-*GOGAT1* in rice [33], as well as the asparagine synthetase *ASN1* and the glutamine synthetase *GLN1* in Arabidopsis [34, 35]. In addition to feedback regulation, glutamine has been proposed to play a role in nitrogen signaling responsible for the regulation of nitrate uptake [31, 32]. Recent work showed that glutamine plays a crucial role in salicylic acid-associated redox status and disease resistance in Arabidopsis [36]. In rice, glutamine has been implicated in the regulation of nitrogen and cytokinin biosynthesis [37].

As one of the major forms of nitrogen in rice [38], glutamine may have functions that go beyond that of a metabolic fuel or precursor for protein synthesis. Here, we demonstrate that glutamine can effectively support the growth of rice seedlings at a relatively low concentration. To test whether glutamine functions as a signaling molecule in plants, we used transcriptome analysis to identify genes that are rapidly regulated by glutamine in rice roots.

## Methods

### Plant material and growth conditions

Rice (*Oryza sativa* L. ssp. japonica cv. TNG67) was used in all experiments. For growth analysis, seeds were germinated in darkness at 30 °C for 3 days and then transferred to hydroponic solutions [39] consisting of modified nitrogen sources, with or without 1.43 mM NH_4_NO_3_, or supplemented with 0.1 to 10 mM glutamine, and grown under controlled conditions (30 °C, 12-h photoperiod, 200 μmol photons m^−2^ s^−1^, 70 % relative humidity) for 2 weeks. The hydroponic solution was renewed every 3 days in all experiments.

### Determination of chlorophyll content

Leaves from 17-day-old rice seedlings grown in hydroponic solutions with 1.43 mM NH_4_NO_3_ (+N), without nitrogen (−N) or with 0.1 to 10 mM glutamine were used for determination of chlorophyll content with a Chlorophyll Content Meter (CCM-300, Opti-sciences, NH, USA).

### RNA isolation and microarray analysis

For microarray analysis, 17-day-old rice seedlings grown in hydroponic solution without NH_4_NO_3_ (−N) were transferred to fresh −N solution or –N solution supplemented with 2.5 mM glutamine (+ Gln) for 30 min. Roots and shoots were harvested separately for total RNA isolation using phenol extraction, as previously described [40]. RNA samples from two biological repeats were sent to the Affymetrix Gene Expression Service Lab at Academia Sinica, Taipei, Taiwan (http://ipmb.sinica.edu.tw/affy/) for target preparation, and hybridization to the GeneChip Rice Genome Array (Affymetrix, Santa Clara, CA, USA). The standard washes and double-staining were performed on the Affymetrix GeneChip Fluidics Station 450, and the arrays were scanned on the Affymetrix GeneChip Scanner 3000. The scanned arrays were analyzed by Affymetrix GCOS version 1.4 and the raw data were saved as CEL files. The GeneSpring GX 11.5 software was used to analyze the microarray data. Unpaired *t*-tests were performed to examine the reproducibility of the data. Two-fold cutoff and a *P*-value less than 0.05 were applied to select for up- and down-regulated genes after 2.5 mM Gln treatment for 30 min. These microarray gene expression data have been deposited in the GEO repository (accession number: GSE56770).

### RT-PCR analysis of glutamine-responsive genes

Seventeen-day-old rice seedlings grown in –N hydroponic solution were transferred to solutions containing 2.5 mM glutamine for 0, 15, 30 min, 1, 4, and 24 h. Total RNA extracted from Gln-treated roots was used for RT-PCR analysis of the glutamine-responsive genes identified by microarray. The RNA samples were digested with DNase I, and reverse transcription was performed with Superscript III RT (Invitrogen, Carlsbad, CA) according to the manufacturer’s instructions. The oligo(dT)_20_ primer was used in reverse transcription to synthesize the first strand cDNA. Taq DNA polymerase and PCR kits were purchased from Roche Diagnostics (Indianapolis, IN). The total volume of a single PCR amplification was 50 μl. For gel electrophoresis, 10 μl of the PCR product was loaded on a 1 % agarose gel. Primers used for cDNA amplification of the 35 glutamine-responsive genes and the control gene *EF1α* are listed in Additional file 1: Table S1.

### Quantitative RT-PCR analysis of glutamine-responsive transcription factor genes

Seventeen-day-old rice seedlings grown in −N hydroponic solution were subsequently transferred to solutions containing 2.5 mM glutamine, glutamate, or 1.43 mM NH_4_NO_3_ for 0, 15, 30 min, 1, 4, and 24 h. Total RNA extracted from roots was digested with DNase I and used for quantitative RT-PCR analysis. The sequences of the primers used for quantitative RT-PCR analysis of glutamine-responsive transcription factor genes are listed in Additional file 1: Table S2. All of the quantifications were normalized to the nuclear gene *UBC3* (*Os02g0634800*). The quantitative RT-PCRs were performed in triplicate for each sample in three independent experiments.

### Amino acid analysis

Seventeen-day-old rice seedlings grown in hydroponic solution without NH_4_NO_3_ (−N) were transferred to fresh −N or −N supplemented with 2.5 mM glutamine (+Gln) for 30 min or the indicated time. Roots and shoots were harvested separately for extraction of free amino acids. Amino acids were extracted with 50 % (*v*/*v*) methanol: H_2_O solution spiked with internal standard 50 μM Norvaline as described [41]. Free amino acids were derivatized with the AccQ•Tag Ultra derivatization kit (Waters, Milford, MA, USA); for derivatization, 10 μL of rice extract was mixed with 70 μL of AccQ•Tag Ultra borate buffer and 20 μL of AccQ•Tag Ultra reagent, followed by incubation for 15 min at 55 °C. Liquid chromatography of the derivatized amino acids was performed on the Waters Acquity UPLC system equipped with a Waters AccQ•Tag Ultra column (2.1 × 10 mm, 1.7 μm particles). The separation gradient used was: 0–0.54 min (99.9 % A), 5.0 min (90.9 % A), 8.50 min (78.8 % A), 8.90–9.50 min (40.4 % A), 9.60–10.10 min (99.9 % A). The eluent A was 10 % AccQ•Tag Ultra concentrate solvent A, eluent B was 100 % acetonitrile. The mobile phase flow rate was 0.7 mL/min, and the sample injection volume was 1 μL. The results shown in Fig. 2 and Table 2 were derived from four biological repeats.

## Results

### Glutamine is an effective nitrogen source for plant growth

**Fig. 1:**
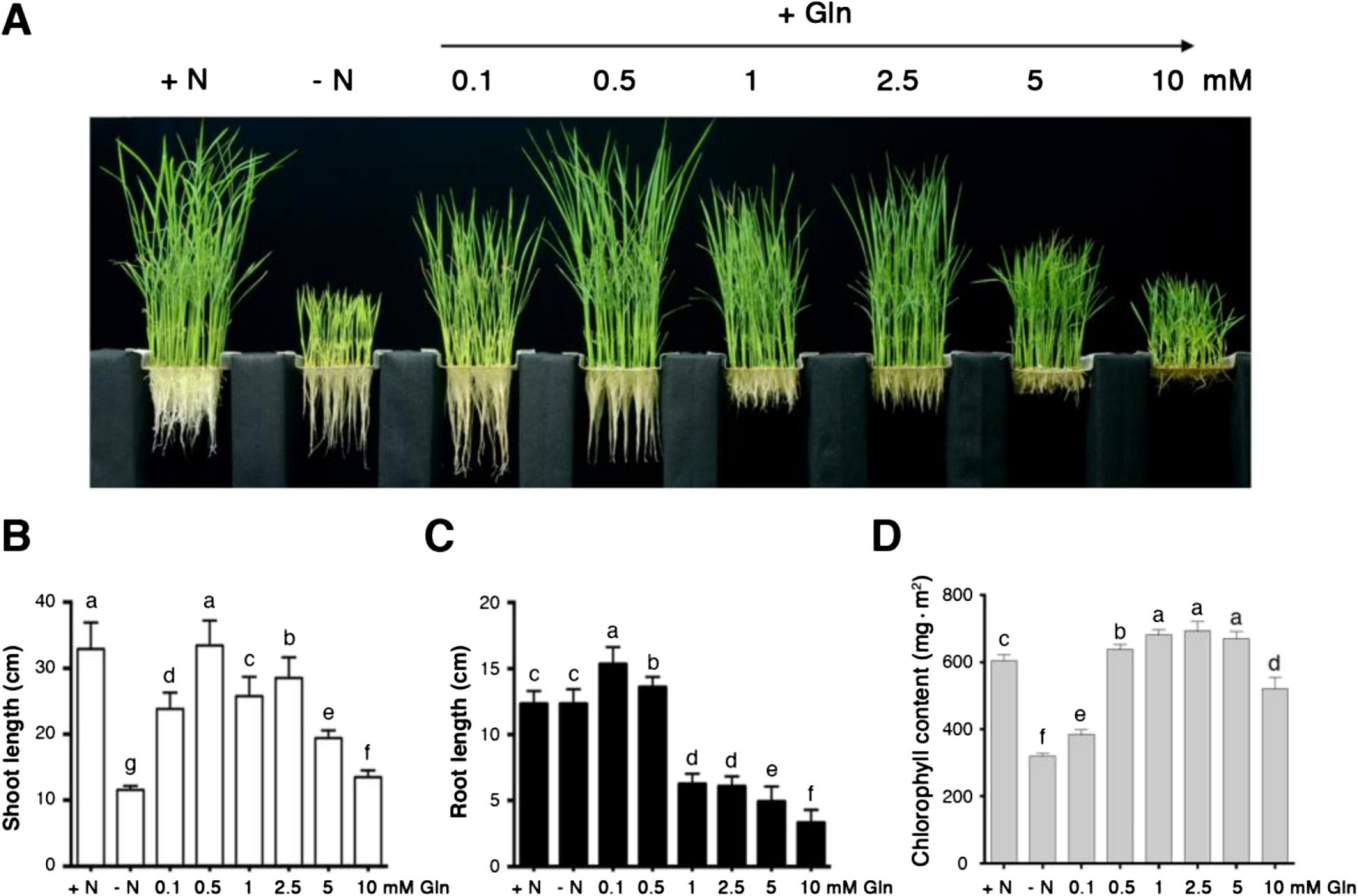
Effects of glutamine on the growth of rice seedlings. **a** 17-day-old rice seedlings grown in hydroponic solutions containing NH_4_NO_3_ or glutamine as the nitrogen source. Shoot length (**b**), root length (**c**), and chlorophyll contents (**d**) of 17-day-old rice seedlings grown in different concentrations of glutamine. Data are means ± SD (*n* = 40). Different *letters* indicate significant differences between treatments, tested by one-way ANOVA followed by Tukey’s test (*P* < 0.05). +N, + 1.43 mM NH_4_NO_3_; −N, no nitrogen

In primary nitrogen assimilation, inorganic nitrogen such as nitrate or ammonium is converted into glutamine and glutamate in plants. However, this is not the only route that plants can use to acquire nitrogen from the environment. Plants can also directly take up amino acids to support their growth and development. We used hydroponic solutions to examine the effects of glutamine as the sole nitrogen source on the growth of rice seedlings (Fig. 1). Compared with seedlings grown in the absence of nitrogen, the addition of 0.1 mM glutamine significantly improved the growth of rice seedlings (Fig. 1a-c). However, the chlorophyll content was low in these seedlings, indicating that rice seedlings grown in 0.1 mM glutamine were still nitrogen deficient (Fig. 1d). When rice seedlings were grown in 0.5 mM glutamine, the length of shoots and roots, and the chlorophyll content were comparable to those grown in 1.43 mM NH_4_NO_3_ (Fig. 1b-d). However, the addition of 1, 2.5, 5, or 10 mM glutamine in the hydroponic solution inhibited the growth of rice seedlings (Fig. 1a-c) and the growth of roots was more sensitive to glutamine inhibition, compared with the growth of shoots. Although the growth of rice seedlings was inhibited by 1, 2.5 and 5 mM glutamine, the chlorophyll content in these seedlings was similar to that of seedlings grown in 1.43 mM NH_4_NO_3_ (Fig. 1d). These results indicated that the inhibitory effects of 1–10 mM glutamine on the growth of rice seedlings were not due to nitrogen deficiency. Similarly, other amino acids such as alanine and glycine could also serve as efficient nitrogen sources for rice seedlings when supplemented in optimal concentrations in the hydroponic system (Additional file 1: Figure S1). These results are consistent with the notion that plants have the capacity to take up amino acids as nitrogen sources [5].

### Nitrogen-starved rice seedlings can rapidly take up glutamine

**Fig. 2:**
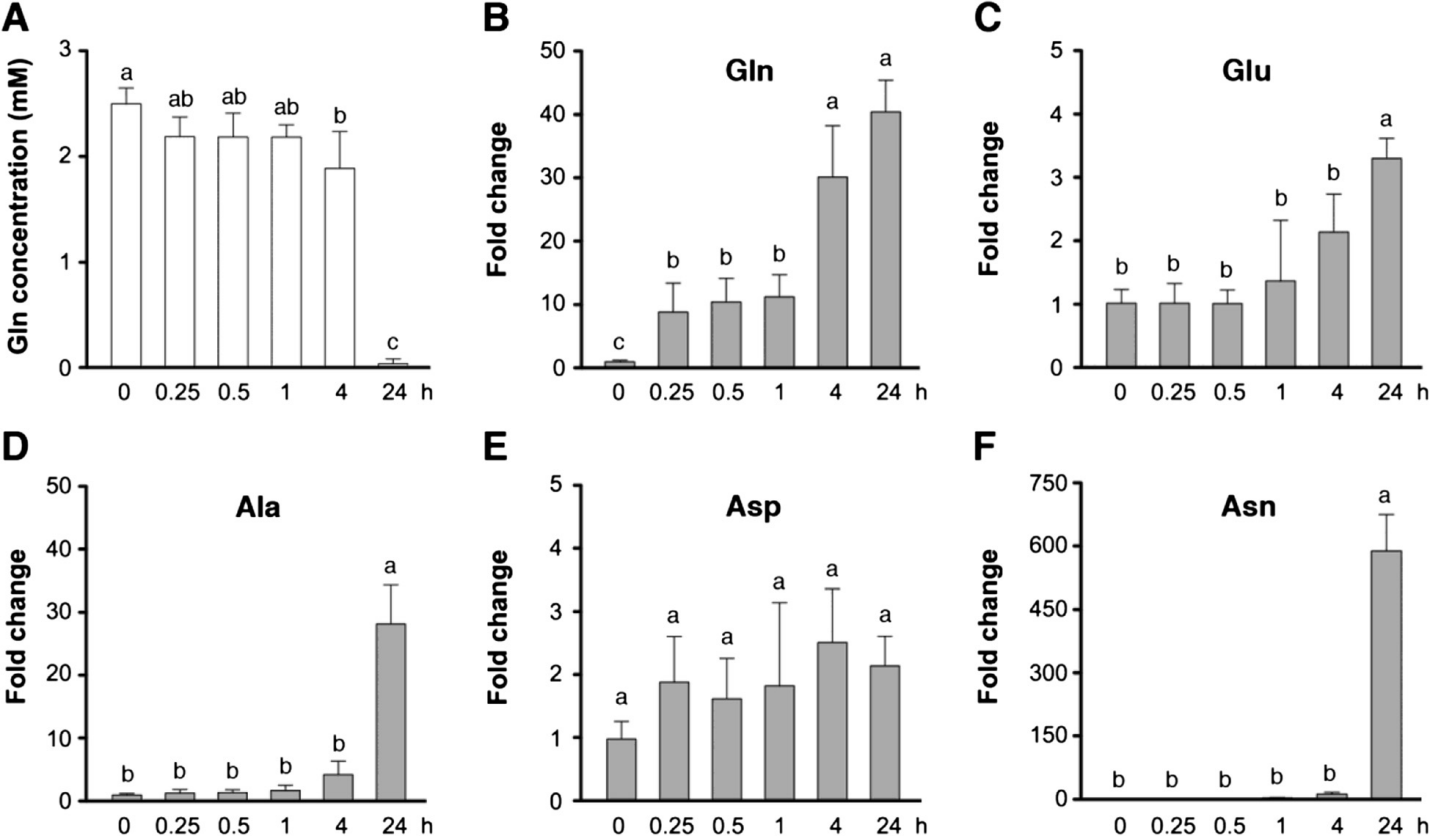
Amino acid contents in the medium and in rice roots during the time course of glutamine treatment. **a** The hydroponic solution initially contained 2.5 mM glutamine and glutamine concentrations were measured over the 24-h time course. **b**-**f** Contents of Gln, Glu, Ala, Asp and Asn in the roots after 0–24 h of glutamine treatment. Fold change indicates the relative amounts of amino acids in 2.5 mM glutamine-treated roots (0.25–24 h) compared to those of control (0 h). Data are means ± SD (*n* = 4). Different *letters* indicate significant differences between treatments, tested by one-way ANOVA followed by Tukey’s test (*P* < 0.05)

In addition to long-term treatments, we also examined if nitrogen-starved rice seedlings could effectively take up glutamine. Seventeen-day-old nitrogen-starved rice seedlings were transferred to hydroponic solutions containing 2.5 mM glutamine for 0–24 h. We then measured the amount of glutamine left in the hydroponic solution during the time course of glutamine treatment. The amount of glutamine in the medium decreased rapidly after 0.25–4 h, and was almost exhausted after 24 h of treatment (Fig. 2a). These results suggested that the rice seedlings could effectively consume glutamine in the hydroponic solution. We further analyzed the levels of free amino acids in the roots during the time course of glutamine treatment. Compared with the nitrogen-starved seedlings, in the glutamine-treated plants, the levels of glutamine, glutamate, alanine, aspartate, and asparagine increased significantly after 24 h of glutamine treatment (Fig. 2b-f). The amount of glutamine increased ~10-fold after 0.25–1 h, ~30-fold after 4 h, and continued to increase to ~40-fold of control levels after 24 h of glutamine treatment (Fig. 2b).

Feeding of glutamine to nitrogen-starved rice seedlings did not significantly increase the amount of glutamate within the first hour, but the levels of glutamate in the roots increased to about 2- to 3-fold of control levels after 4–24 h of glutamine treatment (Fig. 2c). Alanine levels increased slightly during the first 4 h, and then increased dramatically to about 30-fold of control after 24 h of glutamine treatment (Fig. 2d). By contrast, the amount of aspartate increased to ~1.5–2.5-fold of control levels during the glutamine treatment but the differences were not statistically significant (Fig. 2e). Asparagine levels were low in nitrogen-starved seedlings, and feeding of glutamine for 0.25–1 h did not increase the amount of asparagine in the roots. However, the amount of asparagine dramatically increased to about 600-fold of control levels after 24 h of glutamine treatment (Fig. 2f).

### Transcriptome analysis of early glutamine-responsive genes in rice roots

In addition to metabolic and nutritional effects, we suspected that glutamine might function as a signaling molecule to rapidly regulate gene expression in rice. To examine this, we treated rice seedlings with 2.5 mM glutamine for 30 min and used microarray analysis to identify genes that were rapidly induced by glutamine. We used 2.5 mM glutamine, a concentration comparable to the concentration of inorganic nitrogen (1.43 mM NO_3_^−^ and 1.43 mM NH_4_^+^) commonly used in hydroponic solution [39]. In roots, 41 genes (2-fold cutoff), including 39 up- and 2 down-regulated by glutamine, were identified in the microarray analysis. However, we were unable to verify the up-regulation of 4 genes (*Os06g0598500*, +2.1-fold; *Os06g0257600*, +2.1-fold; *Os05g0427800*, +2.2-fold; mitochondrial *rps1*, +2.6-fold), and the down-regulation of 2 genes (*Os04g0607500*, −2.3-fold; *Os06g0695500*, −2.4-fold) by RT-PCR (data not shown). Thus, we concluded that glutamine rapidly upregulated the expression of at least 35 genes in rice roots (Table 1). The functions of these genes are related to metabolism, transport, signal transduction, and stress responses.

**Table 1:** Glutamine-responsive genes in rice roots

Locus ID	Accession no.	Fold change (+ Gln/− N)	Gene description
Os05g0114400	NM_001060995	5.4	ZOS5-02, C2H2 zinc finger protein
Os09g0522200	NM_001070247	4.0	DREB1A, dehydration-responsive element-binding protein 1A
Os11g0184900	NM_001072451	4.0	NAC5, no apical meristem protein 5
Os03g0236200	NM_001056025	3.6	GAD3, glutamate decarboxylase 3
Os06g0633100	NM_001064664	3.6	GDU-like, glutamine dumper-like
Os03g0823400	NM_001058277	3.5	BBTI13, Bowman-Birk type bran trypsin inhibitor 13
Os09g0482800	NM_001188974	3.2	EF hand family protein
Os01g0952900	NM_001051958	3.1	IRO2, helix-loop-helix DNA-binding domain containing protein
Os08g0386200	NM_001068242	3.0	WRKY69
Os05g0545300	NM_001062746	2.8	MAPKKK57
Os04g0673300	NM_001060766	2.8	RR6, type-A response regulator 6
Os08g0172900	N.A.	2.8	Unknown protein
Os03g0445700	NM_001057003	2.7	LBD37-like
Os08g0138100	N.A.	2.7	Uclacyanin 1 (UCC1)-like
Os07g0410300	NM_001188228	2.6	AP2/ERF106, AP2 domain containing protein
Os02g0687200	NM_001054310	2.6	DUF581, domain of unknown function 581
Os07g0119300	NM_001065305	2.6	MYB-like family transcription factor
Os03g0564600	NM_001057032	2.6	RLK-like
Os05g0194900	NM_001061394	2.4	PFK04, phosphofructokinase 04
Os01g0975000	NM_001052095	2.4	DUF966, domain of unknown function 966
Os02g0532900	NM_001053562	2.4	GH17, glycosyl hydrolases family 17
Os05g0402900	NM_001062031	2.4	EDGP-like, xylanase inhibitor
Os01g0699100	NM_001050512	2.3	MAPKKK63
Os07g0589000	NM_001066683	2.3	LBD37-like
Os12g0113500	NM_001072509	2.3	CIPK14, calcineurin B-like interacting protein kinase 14
Os02g0585100	NM_001053799	2.3	Heavy metal associated (HMA) domain containing protein
Os08g0138200	NM_001067493	2.2	Uclacyanin 1 (UCC1)-like
Os04g0301500	NM_001058941	2.2	bHLH, basic helix-loop-helix family protein
Os02g0205500	NM_001052785	2.2	KCS11, 3-ketoacyl-CoA synthase 11
Os03g0187800	NM_001055748	2.2	PUP3-like, purine permease 3-like
Os02g0807900	NM_001054992	2.1	WAK21, cell wall associated kinase 21
Os04g0194500	NM_001058746	2.1	ABC transporter
Os01g0845000	NM_001185719	2.0	DUF668, domain of unknown function 668
Os08g0446800	NM_001068463	2.0	GDU-like, glutamine dumper-like
Os03g0124800	NC_008396	2.0	Unknown protein

Total RNA extracted from 17-day-old rice seedlings grown in hydroponic solution without nitrogen (−N) or treated with 2.5 mM glutamine for 30 min (+ Gln) was used for microarray analysis. The results were derived from two biological replicates. Os08g0172900 and Os08g0138100 were annotated in the RIKEN BASE (https://database.riken.jp/sw/en/RIKENBASE/rib158i/) but not available (N.A.) in National Center for Biotechnology Information (http://www.ncbi.nlm.nih.gov/)

Interestingly, 10 of the 35 early glutamine-responsive genes encode putative transcription factors, including two LBD37-like proteins, Os03g0445700 and Os07g0589000 (Table 1). Arabidopsis LBD/37/38/39 transcription factors are involved in the regulation of nitrogen responses [42]. Rice has two LBD37-like proteins, Os03g0445700 and Os07g0589000 (http://www.ncbi.nlm.nih.gov/homologene/?term=Arabidopsis%20LBD37), which share 72 % amino acid sequence similarity to each other (Additional file 1: Figure S2). Although the exact functions of rice LBD37-like proteins are still unknown, the LBD37-like proteins, Os03g0445700, has been shown to be associated with nitrogen metabolism in rice [43]. The other glutamine-responsive transcription factor genes are: *ZOS5*-*2* (*Os05g0114400*), a C2H2 zinc finger protein [44]; *DREB1A* (*Os09g0522200*), dehydration-responsive element-binding protein 1A involved in cold signaling [45]; *NAC5* (*Os11g0184900*), no apical meristem protein 5 involved in abiotic stress responses [46–48]; *IRO2* (*Os01g0952900*), a helix-loop-helix DNA-binding domain containing protein involved in iron uptake under iron deficiency [49–51] and GA3 responses [52]; *WRKY69* (*Os08g0386200*), a WRKY domain-containing protein; *AP2*/*ERF106* (*Os07g0410300*), an AP2/ERF domain-containing protein [53]; *MYB*-*like* (*Os07g0119300*), a MYB-like family protein; and *bHLH* (*Os04g0301500*), a basic helix-loop-helix family protein.

In addition to transcription factor genes, the early glutamine-responsive genes included several genes encoding proteins involved in various signaling pathways. The calcineurin B-like interacting protein kinase 14 (CIPK14, Os12g0113500) is involved in the regulation of defense and stress signaling [54, 55]. The *RR6* (*Os04g0673300*) gene encodes a type-A response regulator that is involved in cytokinin signaling pathways [56, 57]. The *Os09g0482800* gene encodes a putative calcium-binding EF-hand family protein. The expression of some kinase genes, including *MAPKKK57* (Os05g0545300), *MAPKKK63* (*Os01g0699100*) [58], *RLK*-*like* (*receptor*-*like kinase*, *Os03g0564600*), and *WAK21* (*cell wall*-*associate kinase 21*, *Os02g0807900*) [59] was also rapidly induced by glutamine (Table 1).

Among the early glutamine-responsive genes, *GAD3* (glutamate decarboxylase 3), two *glutamine dumper* (*GDU*)-*like* genes (*Os06g0633100* and *Os08g0446800*), *PFK04* (*phosphofructokinase 04*), *glycosyl hydrolase family 17* (*GH17*, *Os02g0532900*), *KCS11* (*3*-*ketoacyl*-*CoA synthase 11*, *Os02g0205500*), *PUP3*-*like* (*purine permease 3*-*like*, *Os03g0187800*), and an ABC transporter gene *Os04g0194500* are involved in metabolism or transport. The expression of several stress response-related genes including *Bowman*-*Birk type bran trypsin inhibitor 13* (*BBTI13*, *Os03g0823400*), *uclacyanin 1* (*UCC1*)-*like* genes (*Os08g0138100* and *Os08g0138200*) encoding plastocyanin-like domain containing proteins, *Os05g0402900* encoding an extracellular dermal glycoprotein (EDGP)-like protein or xylanase inhibitor, and *Os02g0585100* encoding a **h**eavy **m**etal **a**ssociated (HMA) domain containing protein, and several unknown function genes (*Os01g0845000*, *Os01g0975000*, *Os02g0687200*, *Os03g0124800*, and *Os08g0172900*) was also rapidly induced by glutamine (Table 1).

In contrast to roots, our microarray analysis did not identify any early glutamine-responsive genes in shoots (2-fold cutoff, data not shown). This is consistent with our finding that the glutamine levels did not change significantly in shoots after 30 min of glutamine treatment (Table 2). Analysis of free amino acids in glutamine-treated (2.5 mM, 30 min) samples revealed that the endogenous levels of glutamine increased dramatically (~11.6-fold) in roots but not in shoots as compared with those in nitrogen-starved rice seedlings (Table 2).

**Table 2:** Amino acid contents (nmole/g FW) of 17-day-old rice seedlings

	Roots			Shoots		
	− N	+ Gln	+ Gln/ − N (fold change)	− N	+ Gln	+ Gln/ − N (fold change)
Glu	550.9 ± 52.9	669.3 ± 83.8	1.22 ± 0.17	1243.9 ± 280.7	1126.5 ± 205.5	0.93 ± 0.21
Asp	252.7 ± 39.0	350.8 ± 51.8	1.40 ± 0.21*	281.0 ± 60.3	228.0 ± 66.0	0.82 ± 0.18
Ser	171.7 ± 22.3	181.4 ± 17.2	1.06 ± 0.10	874.7 ± 224.9	666.0 ± 164.1	0.77 ± 0.16
Gln	108.1 ± 15.0	1275.5 ± 397.8	11.62 ± 2.36*	622.9 ± 302.7	469.0 ± 262.3	0.75 ± 0.21
Ala	81.1 ± 11.7	142.9 ± 24.7	1.76 ± 0.21*	1063.5 ± 297.9	847.3 ± 183.3	0.81 ± 0.11
Val	46.5 ± 6.8	47.9 ± 5.5	1.04 ± 0.15	225.7 ± 64.7	160.4 ± 38.3	0.73 ± 0.12
Cys	44.9 ± 11.0	46.8 ± 4.7	1.08 ± 0.19	340.7 ± 96.2	273.1 ± 81.3	0.80 ± 0.07
Leu	36.9 ± 6.9	35.2 ± 4.4	0.98 ± 0.22	60.4 ± 13.0	44.1 ± 9.0	0.74 ± 0.08
Thr	36.2 ± 2.1	39.1 ± 4.1	1.08 ± 0.13	160.0 ± 44.3	120.9 ± 34.7	0.76 ± 0.13
Pro	30.8 ± 13.5	28.4 ± 9.2	0.99 ± 0.30	166.4 ± 83.1	139.4 ± 79.8	0.82 ± 0.09
Ile	28.1 ± 4.5	25.5 ± 2.9	0.93 ± 0.17	49.8 ± 17.9	32.4 ± 7.2	0.68 ± 0.12
Trp	26.8 ± 9.6	26.5 ± 9.6	0.99 ± 0.06	416.7 ± 286.2	297.7 ± 144.1	0.78 ± 0.14
Lys	26.7 ± 5.1	27.4 ± 3.6	1.05 ± 0.19	183.0 ± 144.5	136.1 ± 95.7	0.80 ± 0.10
Tyr	24.9 ± 4.5	29.1 ± 2.0	1.19 ± 0.15	333.9 ± 87.0	264.9 ± 72.7	0.81 ± 0.19
His	24.0 ± 10.6	20.5 ± 5.6	0.91 ± 0.17	609.1 ± 488.3	396.3 ± 289.7	0.68 ± 0.14
Asn	21.6 ± 11.4	21.5 ± 11.2	1.03 ± 0.40	130.7 ± 95.3	119.9 ± 84.8	0.96 ± 0.32
Arg	20.2 ± 4.3	14.8 ± 3.5	0.74 ± 0.14	60.9 ± 16.6	43.6 ± 11.4	0.72 ± 0.12
Phe	14.8 ± 2.4	15.7 ± 3.6	1.06 ± 0.16	378.6 ± 96.2	291.5 ± 61.8	0.78 ± 0.09
Gly	14.6 ± 1.2	14.1 ± 1.9	0.96 ± 0.07	87.6 ± 25.3	67.6 ± 17.8	0.80 ± 0.21
Met	9.0 ± 0.7	10.4 ± 1.0	1.16 ± 0.11	60.4 ± 13.1	44.5 ± 7.5	0.74 ± 0.05

− *N* without nitrogen, + *Gln* treated with 2.5 mM glutamine for 30 min. Results were derived from four biological replicates. *indicates *p* < 0.05 after analysis with Student’s *t*-test

### Verification of the microarray data by RT-PCR

**Fig. 3:**
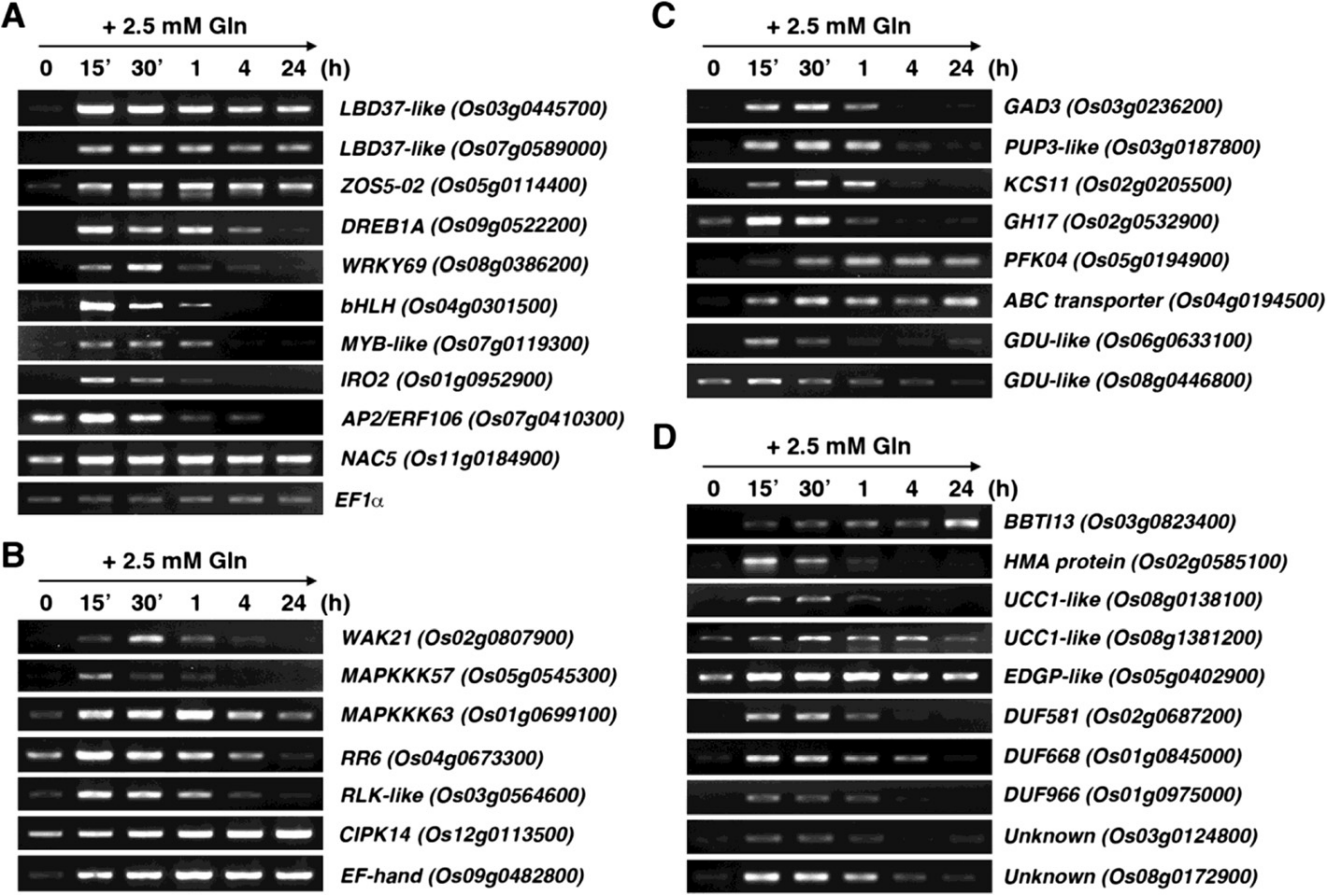
RT-PCR analyses of glutamine-responsive genes. Total RNA extracted from roots of 17-day-old glutamine-treated rice seedlings was used for RT-PCR analysis to verify the expression of 35 genes identified by microarray analysis. **a** Transcription factor genes. **b** Kinase or signal transducer genes. **c** Metabolic or transporter genes. **d** Stress response or unknown function genes. The expression levels of *EF1α* in the same samples are shown as a control

To independently verify the microarray data, we used RT-PCR to examine the effects of glutamine treatments (2.5 mM, 15 min to 24 h) on the expression of all 35 early glutamine-responsive genes in the rice roots. Interestingly, most of the glutamine-responsive genes were rapidly induced by glutamine, increasing within 15 min (Fig. 3). The expression of two *LBD37*-*like* genes and *ZOS5*-*02* was rapidly induced by glutamine and remained at high levels after 4 to 24 h (Fig. 3a). By contrast, the expression of *DREB1A*, *WRKY69*, *bHLH*, *MYB*-*like*, and *IRO2* was rapidly induced by glutamine, peaked between 15 and 60 min, and decreased to levels comparable to those of control after 4 to 24 h (Fig. 3a). Steady-state levels of *AP2*/*ERF106* transcripts increased rapidly and transiently after 15–30 min of glutamine treatment, whereas the expression of *NAC5* was rapidly induced and stayed at high levels during the time course of glutamine treatment (Fig. 3a). The expression of *EF1α* in the same samples was analyzed by RT-PCR as a control (Fig. 3a).

In addition to transcription factor genes, glutamine also rapidly induced the expression of several kinase and regulatory genes. The expression of *MAPKKK57* and *WAK21* was transiently induced by glutamine and peaked at 15 and 30 min, respectively (Fig. 3b). The expression of *MAPKKK63*, *RR6*, and *RLK*-*like* was rapidly induced by glutamine, peaked at 15–60 min, and decreased to levels comparable to those of control after 4–24 h (Fig. 3b). By contrast, steady-state levels of *CIPK14* transcripts increased rapidly and continued to accumulate to higher levels during the time course of glutamine treatment (Fig. 3b). Similarly, the expression of *EF*-*hand* (*Os09g0482800*) was rapidly and strongly induced by glutamine after 15–30 min and remained at high levels after 1–24 h (Fig. 3b).

For metabolic and transporter genes, the expression of *GAD3*, *PUP3*-*like*, *KCS11*, and *GH17* was rapidly induced by glutamine, peaked at 15–60 min, and decreased to levels comparable to those of control after 4–24 h (Fig. 3c). By contrast, the expression of *PFK04* and an ABC transporter gene *Os04g0194500* was continuously induced by glutamine during the time course of glutamine treatment (Fig. 3c). The expression of two *GDU*-*like* genes was rapidly and transiently induced by glutamine after treatments for 15–30 min (Fig. 3c).

The expression of stress-responsive gene *BBTI13* was rapidly and continuously induced by glutamine (Fig. 3d). By contrast, the expression of *HMA* and a *UCC1*-*like* (*Os08g0138100*) gene was only transiently induced by glutamine after 15–30 min (Fig. 3d). The expression of another *UCC1*-*like* gene (*Os08g0138200*) and a stress-responsive *EDGP*-*like* gene was induced by glutamine after 15 min–4 h and decreased to levels comparable to those of control after 24 h (Fig. 3d). The expression of several unknown function genes, including *DUF581*, *DUF668*, *DUF966*, *Os03g0124800*, and *Os08g0172900*, was rapidly induced by glutamine, peaked at 15–30 min, and gradually decreased to levels comparable to those of control after 4–24 h (Fig. 3d).

### Effects of glutamate and ammonium nitrate on the expression of glutamine-responsive transcription factor genes

**Fig. 4:**
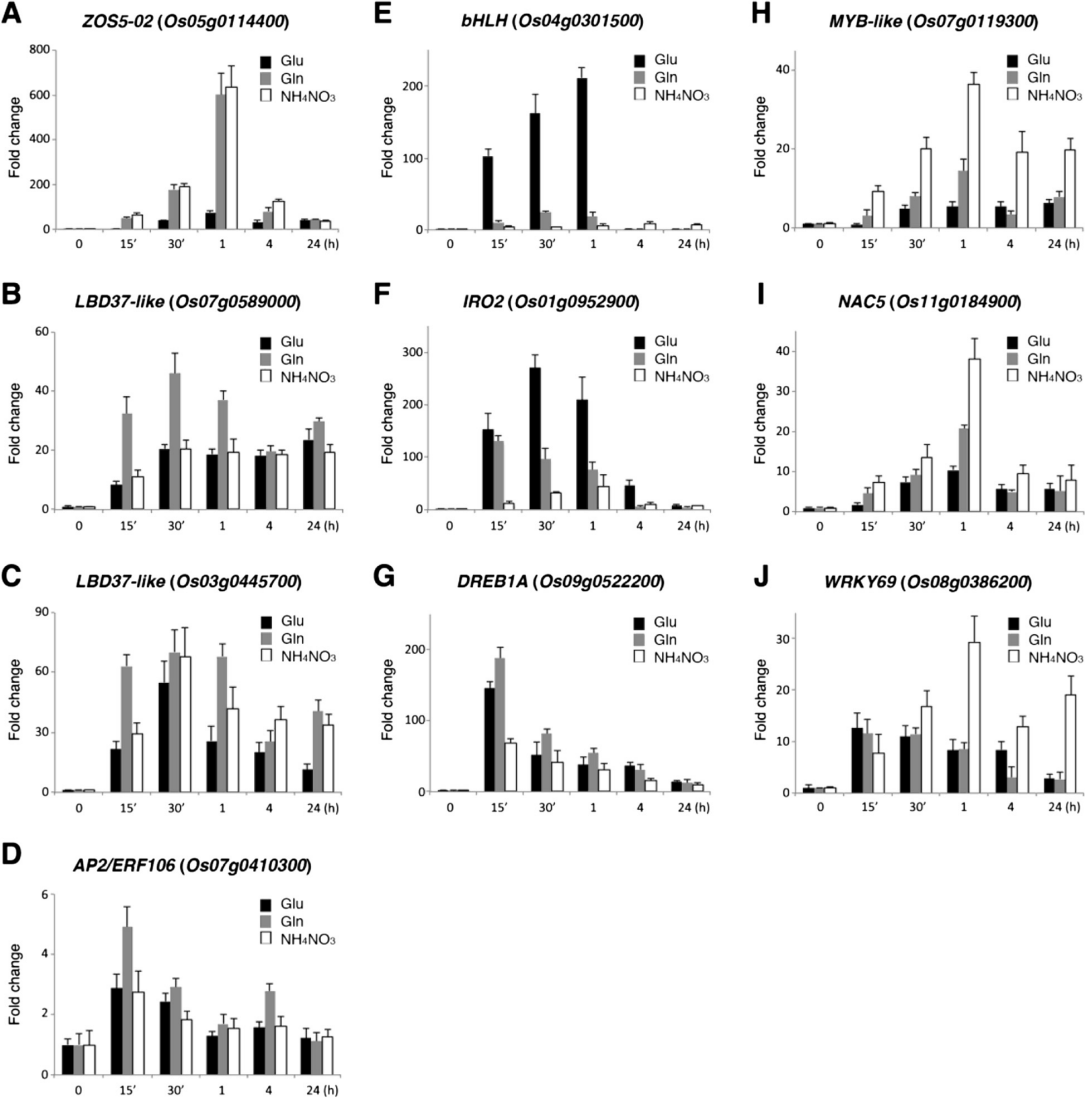
Quantitative RT-PCR analyses of glutamine-responsive transcription factor genes. Seventeen-day-old rice seedlings grown in hydroponic solution without nitrogen were subsequently transferred to medium containing 2.5 mM glutamine, glutamate, or 1.43 mM ammonium nitrate for 0, 15′, 30′, 1, 4, and 24 h. Total RNA extracted from roots was used for quantitative RT-PCR to analyze the expression of *ZOS5*-*02* (**a**), *LBD37*-*like Os07g0589000* (**b**), *LBD37*-*like Os03g0445700* (**c**), *AP2*/*ERF106* (**d**), *bHLH Os04g0301500* (**e**), *IRO2* (**f**), *DREB1A* (**g**), *MYB*-*like Os07g0119300* (**h**), *NAC5* (**i**), and *WRKY69* (**j**). The expression level of each gene in the control sample (0 h) was set at 1. Fold change indicates the relative expression of each gene as compared to that of control. Quantitative RT-PCRs were performed in triplicate for each sample in three independent experiments. All of the quantifications were normalized to the nuclear gene *UBC3* (*Os02g0634800*)

To further examine if the transcriptional response was specific to glutamine, we used quantitative RT-PCR to compare the effects of glutamine, glutamate, and ammonium nitrate on the expression of early glutamine-responsive transcription factor genes. Nitrogen-starved rice seedlings were transferred to hydroponic solutions containing 2.5 mM glutamine, glutamate, or 1.43 mM ammonium nitrate for 15 min to 24 h. Compared with the expression in nitrogen-starved rice seedlings, the expression of these transcription factor genes was also rapidly induced by glutamate and ammonium nitrate in the roots (Fig. 4). However, the expression patterns and the amount of transcripts accumulated in response to different nitrogen sources varied from gene to gene. Some genes were commonly induced by glutamine, glutamate, and ammonium nitrate to similar extents, whereas some genes were preferentially induced by a specific nitrogen source. For instance, the expression of *ZOS5*-*02* was strongly induced by glutamine and ammonium nitrate (~180-fold, 30 min; ~600-fold, 1 h), but induced less strongly by glutamate (38-fold, 30 min; 75-fold, 1 h) (Fig. 4a). Although the expression of *LBD37*-*like* (*Os07g0589000*) was induced by glutamate, glutamine, and ammonium nitrate to similar levels after 4 and 24 h, glutamine seemed to have stronger effects than glutamate and ammonium nitrate on the induction of this gene within the first hour (Fig. 4b). Similarly, glutamine had a stronger effect than glutamate and ammonium nitrate on the induction of another *LBD37*-*like* gene (*Os03g0445700*) and *AP2*/*ERF106* after treatments for 15 min (Fig. 4c,d).

By contrast, the expression of *bHLH* was rapidly and markedly induced by glutamate, which showed a stronger effect than glutamine and ammonium nitrate within the first hour of treatments (Fig. 4e). Similar to *bHLH*, the expression of *IRO2* was rapidly and strongly induced by glutamate within the first hour, whereas the induction by glutamine and ammonium nitrate was relatively mild after treatments for 30 min to 1 h (Fig. 4f). While the expression of *DREB1A* was induced by all nitrogen sources to similar levels after 0.5 to 24 h of treatments, glutamate, and glutamine seemed to have stronger effects than ammonium nitrate on the initial induction (e.g. 15 min) of *DREB1A* (Fig. 4g).

Although all nitrogen sources could rapidly induce the expression of *MYB*-*like*, ammonium nitrate had more pronounced effects than glutamate and glutamine on the induction of *MYB*-*like* during the treatments (Fig. 4h). Similarly, the expression of *NAC5* and *WRKY69* was induced by all nitrogen sources. Nevertheless, ammonium nitrate had stronger effects on the induction of these genes than glutamate and glutamine after 1 h of treatment (Fig. 4i,j).

## Discussion

### Glutamine plays a critical role in plant nutrition and metabolism

Glutamine may enter the plant cell through amino acid transporters and can serve as a critical nitrogen source for plant growth and development. We demonstrated that rice could effectively use glutamine as a nitrogen source at a concentration significantly lower than that of ammonium nitrate. Based on parameters such as root and shoot length, and chlorophyll content, we showed that supplementation of 0.1 mM glutamine could significantly improve rice seedling growth and 0.5 mM glutamine was as effective as 1.43 mM ammonium nitrate (Fig. 1). These results suggest that glutamine is a potential nitrogen source for plants in natural environments. Analysis of amino acid levels after glutamine time course treatment further confirmed that rice could rapidly take up glutamine from hydroponic solution. Interestingly, the sudden increase of glutamine also rapidly induced the expression of two *GDU*-*like* genes (Fig. 3). Glutamine dumpers are plant-specific membrane proteins that are involved in nonselective amino acid export [60]. The Arabidopsis *GDU1* overexpression lines have enhanced glutamine secretion, reduced amino acid uptake, and increased amino acid contents in leaf apoplasm and xylem sap [60, 61]. The induction of *GDU*-*like* genes by glutamine may promote the allocation of amino acids in the apoplast and xylem sap, which may help the transport of amino acids to nitrogen-starved tissues.

In addition to synthesis of proteins and nucleotides, one of the major routes of glutamine metabolism is the formation of glutamate catalyzed by Fd- or NADH-GOGAT in plants. In glutamine-treated rice roots, some of the excess glutamine may be quickly converted to glutamate. However, the amounts of glutamate did not change significantly during the first 4 h, and only increased slightly after 24 h of glutamine treatment (Fig. 2c). Glutamate is a very active amino acid that may be rapidly converted to other nitrogen-containing compounds in the cell. For instance, glutamate is the primary nitrogen donor for most transamination reactions in plants [6]. In nitrogen-deficient cells, glutamate may donate its amine group to α-keto acids such as pyruvate and oxaloacetate for the synthesis of alanine and aspartate, respectively. In fact, the amounts of alanine and aspartate increased rapidly in glutamine-treated roots (Table 2).

In contrast to glutamate, asparagine is a relatively inert amino acid. Asparagine started to accumulate in glutamine-treated roots after 4 h and increased dramatically after 24 h. It is possible that the rice seedlings were no longer deficient of nitrogen after 4–24 h of glutamine treatment. Asparagine and glutamine have higher nitrogen to carbon ratios and usually serve as nitrogen storage or transport compounds in plants. Thus, the plants may have stored excess nitrogen as asparagine and glutamine in 24 h glutamine-treated roots (Fig. 2). By contrast, plant cells have to balance nitrogen metabolism by maintaining the homeostasis of glutamate [6]. So the content of glutamate will not fluctuate dramatically, even in excess-nitrogen conditions.

### Glutamine rapidly regulates gene expression in plants

In addition to glutamine’s role as a metabolic fuel, increasing evidence has implicated that glutamine sensing and signaling pathways also exist in plants [14]. Besides its well-known effects on the activation of mTORC1 signaling pathways, glutamine also affects the expression of several key transcription factor genes involved in stress responses in humans [16, 17]. It is intriguing that 10 of the 35 early glutamine-responsive genes identified in this study encode putative transcription factors. Notably, glutamine rapidly induced two *LBD37*-*like* genes that regulate nitrogen responses in plants [42, 43] (Fig. 3). In addition, *DREB1A*, *IRO2*, and *NAC5* encoding transcription factors involved in various stress responses [45–51] are among the early glutamine-responsive genes identified in this study. It is not clear why glutamine activates the expression of these genes. One of the possible explanations is that these stress-related transcription factors also function in the regulation of nitrogen responses in rice. Alternatively, the glutamine signaling pathway may link to a number of cell functions including stress tolerance beyond nitrogen metabolism via the activation of these transcription factors in plants. Of course, further studies are required to explore these possibilities. The functions of the other early glutamine-responsive transcription factor genes, including *ZOS5*-*02*, *WRKY69*, *bHLH* (*Os04g0301500*), *MYB*-*like* (*Os07g0119300*), and *ERF106* (*Os07g0410300*), have yet to be characterized. It will be interesting to examine if these putative transcription factors are involved in the interplay of nitrogen and stress signaling pathways in rice.

In addition to transcription factor genes, the expression of several kinase genes, including *CIPK14*, *MAPKKK57*, *MAPKKK63*, *WAK21*, and *RLK*-*like*, which function in various signaling pathways, was also rapidly induced by glutamine. Calcium (Ca^2+^) is a universal second messenger that plays an important role in many signaling pathways in plants. Calcineurin B-like proteins are Ca^2+^ sensors that specifically interact with CIPKs in plant Ca^2+^ signaling. Interestingly, glutamine rapidly induced the expression of *CIPK14* (Fig. 3), which functions in biotic and abiotic responses [54, 55]. Similarly, the expression of an *EF*-*hand* gene encoding a putative Ca^2+^-binding protein was also rapidly induced by glutamine (Fig. 3). It is possible that the multifunctional Ca^2+^ signaling pathways are involved in glutamine sensing and signaling in plants. It will be interesting to examine if CIPK14 also plays a role in integrating nitrogen responses to stress signaling pathways in rice. The functions of *MAPKKK57*, *MAPKKK63*, *WAK21*, and *RLK*-*like* genes have yet to be characterized. Further studies are required to examine if these kinases are involved in glutamine or nitrogen signaling pathways in plants.

The plant hormone cytokinins have been closely linked to nitrogen signaling [62]. Glutamine-related signals are involved in the regulation of cytokinin biosynthesis [37]. Response regulators (RR) act as important components of the cytokinin signaling pathways in Arabidopsis [63]. In addition, transport of cytokinins mediated by purine transporters of the PUP family is involved in long-distance signaling [64]. Interestingly, glutamine rapidly induced the expression of *RR6* and *PUP3*-*like* genes in rice roots (Fig. 3). These results suggest that glutamine may exert its functions by directly interacting with cytokinin biosynthesis and signaling pathways. It will be interesting to further examine if glutamine can trigger local and long distance cytokinin signaling via the induction of *RR6* and *PUP3*-*like* genes in rice.

### Crosstalk between glutamine and stress signaling pathways in plants

In addition to *DREB1A*, *IRO2*, *NAC5*, and *CIPK14*, glutamine also rapidly induced the expression of many stress-related genes, such as *EDGP*-*like*, *BBTI13*, *HMA*, *UCC1*-*like*, *KCS11*, and *GH17* (Fig. 3). These results are reminiscent of the glutamine studies in humans. Glutamine is the most abundant amino acid in humans and its function goes beyond that of a fuel for metabolism or a precursor for protein synthesis [16]. A number of studies reveal that glutamine can regulate the expression of a large variety of target genes involved in major functions including stress responses [16, 17]. In addition to the regulation of metabolism-related genes, glutamine also induces a diverse array of transcription factor genes involved in inflammatory responses, proliferation, apoptosis, and survival in mammals [16, 17]. Despite its central regulatory role in numerous functions, the underlying molecular mechanisms and most of the signaling pathways remain to be identified. Similarly, glutamine is one of the most abundant amino acid in plants. Besides its role as metabolic fuel and a precursor of proteins, nucleic acids, and amino sugars, glutamine may have additional functions that have yet to be established in plants.

The induction of stress-responsive genes by glutamine raises an interesting question whether glutamine, an important nutrient, also functions as a stress signal in plants. Generally, if the amount of nutrients exceeds the amount required, this excess may inhibit normal growth, development, and metabolism. Consistent with this notion, we have shown that high concentrations of amino acids can inhibit the growth of rice seedlings. When the model plant Arabidopsis was grown on medium containing 5 mM glutamine as the sole nitrogen source, the biomass was comparable to that of plants grown on nitrate [65]. By contrast, growth on 1 mM glutamine significantly inhibited root growth in rice (Fig. 1). While the mechanism of this amino acid inhibition is not well understood, rice seems to be more sensitive to glutamine inhibition than Arabidopsis. It is well known that most of the amino acid biosynthetic pathways are regulated by feedback inhibition. It is possible that glutamine in excess may inhibit some enzymes or interfere the absorbance of other nutrients. Thus, the specific growth condition with high concentrations of single amino acids, e.g. glutamine in this study, may cause growth inhibition and trigger the expression of stress-response genes.

The effects of glutamine on gene expression may occur by a direct effect of glutamine or through products of glutamine metabolism. Although the mechanism by which glutamine affects gene expression is not well understood, the synthesis of glutathione derived from glutamine, and the increase of reactive oxygen species (ROS) after glutamine addition are among the possible mechanisms that mediate the glutamine responses in mammals [16]. It will be interesting to examine if glutamine changes the redox status or affects the production of ROS in plants. Most stress-responsive genes, including *DREB1A*, *IRO2*, *HMA*, *UCC1*-*like*, *KCS11*, and *GH17*, and many other genes identified in this study, were rapidly and transiently (15–60 min) induced by glutamine (Fig. 3). It is possible that the sudden and dramatic increase of endogenous levels of glutamine may affect the cell’s redox status and ROS production, which in turn may induce the expression of stress-responsive genes.

Regardless of the underlying molecular mechanisms, the effects of glutamine on the regulation of gene expression suggest that glutamine may interact with stress signaling pathways in plants. While amino acids such as glutamine can serve as nutrients to support plant growth and development, they may also function as stressors to elicit defense responses in plants.

### Regulation of glutamine-responsive genes by general nitrogen status

The expression of most glutamine-responsive transcription factor genes was also rapidly induced by glutamate and ammonium nitrate (Fig. 4). These results suggest that the expression of these genes is not specifically regulated by glutamine. The transition from nitrogen deficiency to nitrogen sufficiency, rather than specific induction by glutamine, may explain the expression patterns of some early glutamine-responsive genes identified in this study. Since the endogenous levels of glutamine continued to increase after 4–24 h of treatment (Fig. 2), it is reasonable to expect that the expression of glutamine-regulated genes would also be maintained at high levels during this time. However, the expression of many glutamine-responsive genes increased significantly within the first hour and then decreased to levels comparable to those of control (Fig. 3). Although this expression pattern does not parallel the endogenous glutamine levels, it is consistent with the notion that the rice seedlings are no longer deficient of nitrogen after 4–24 h of glutamine treatment, as indicated by the dramatic accumulation of asparagine and glutamine (Fig. 2). Thus, signals derived from overall nitrogen deficiency or nitrogen sufficiency, rather than a specific nitrogen source, may play a major role in the regulation of these genes.

In addition to sensing endogenous levels of nitrogen, plants may also have mechanisms to sense the nitrogen status in the environment. For instance, nitrate transporters have been proposed to function as nitrate sensors in plants [66]. It is conceivable that similar sensing mechanism for glutamine or the availability of nitrogen may exist in plants. Rice seedlings may be able to sense the exogenous levels of glutamine or the availability of nitrogen and respond with specific regulation of gene expression. The expression patterns of some glutamine-responsive genes, for instance, those involved in signal transduction including *DREB1A*, *MAPKKK63*, *RR6* and *RLK*-*like*, correspond well with the availability of glutamine in the medium. The expression of these genes was induced by glutamine to their highest levels at 15–60 min, was lower at 4 h, and was almost not induced at 24 h (Fig. 3). Regardless of the specific nitrogen source, the combination of internal nitrogen status (e.g. nitrogen deficiency versus nitrogen sufficiency) and the availability of external nitrogen may contribute to the regulation of early glutamine-responsive genes identified here.

Still, the expression levels of glutamine-responsive transcription factor genes were differentially induced by glutamine, glutamate and ammonium nitrate. For instance, glutamine had stronger effects on the induction of *LBD37*-*like* (*Os07g0589000*) than did glutamate and ammonium nitrate. The expression of *bHLH* and *IRO2* is preferentially induced by glutamate, whereas ammonium nitrate has stronger effects on the induction of *MYB*-*like* and *NAC5*. It is likely that the general nitrogen status precedes the specificity of nitrogen source in regulating the expression of these transcription factor genes. Thus, the addition of organic (e.g. glutamine or glutamate) or inorganic nitrogen to nitrogen-starved rice seedlings would generally induce the expression of these genes, but the levels of induction were determined by the specific nitrogen source.

### Amino acid sensing and signaling in plants

Amino acids are biochemically diverse molecules that are essential for cell growth. Although glutamine and glutamate are closely related in metabolism, they may have distinct effects on the induction of plant gene expression. The expression of *ZOS5*-*02* was rapidly and dramatically induced by glutamine, but only mildly induced by glutamate (Fig. 4). By constrast, the expression of *bHLH* was rapidly and strongly induced by glutamate, but only mildly induced by glutamine (Fig. 4). These results imply that different amino acids may have some specificity in the regulation of plant gene expression. However, it remains unclear how plants sense specific amino acids. In mammalian cells, mTORC1 is involved in integrating the availability of nutrients and growth factors to regulate cell growth and proliferation. Several amino acids, including glutamine, arginine, and leucine, effectively activate mTORC1, and intensive studies have just begun to uncover the complex machinery of amino acid sensing by mTORC1 in mammalian cells [20, 67]. It is not clear if similar regulatory systems exist in plants. The largely unexplored amino acid sensing mechanisms in plants are expected to be at least as complicated as those in mammals.

Because nitrate is the primary nitrogen source for plants, most studies on plant nitrogen sensing and signaling have focused on nitrate. Since nitrate can be readily converted into glutamine and glutamate, it is possible that many nitrogen responses triggered by inorganic nitrogen are in fact partly mediated by glutamine or glutamate. It is interesting that plant PII proteins have been recently demonstrated to sense glutamine to regulate the activity of NAGK and the production of arginine and polyamine [14]. The functions of glutamine and the other amino acids likely go beyond that of metabolic regulation in plants. Further studies on the physiological roles and the sensing mechanisms of each amino acid are required to advance our knowledge of these processes in plants.

## Conclusion

**Fig. 5:**
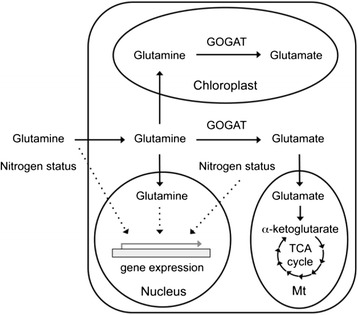
Glutamine may function as a signaling nutrient in plants. Glutamine entering the plant cell can be directly used for metabolism to support plant growth and development. The internal glutamine and nitrogen status may affect gene expression in the nucleus. In addition, plants may be able to sense the external glutamine and nitrogen status to regulate gene expression. *GOGAT* glutamine oxoglutarate aminotransferase, *TCA* tricarboxylic acid, *Mt* mitochondrion

Increasing evidence shows that glutamine, the most abundant free amino acid in humans, plays critical roles in nutrition, metabolism, and signaling in mammals [16]. Glutamine is also one of the most abundant free amino acids in plants, and is important for protein and nucleotide synthesis. Here, we showed that glutamine might play a role in plant nutrition, and potentially function as a signaling molecule to regulate the expression of key transcription factor genes involved in nitrogen and stress responses (Fig. 5). The functions of early glutamine-responsive genes are very diverse, including metabolism, transport, signal transduction, and stress responses. Almost one-third of the early glutamine- responsive genes identified here encode transcription factors, indicating that glutamine may efficiently amplify its signal and interact with the other signal transduction pathways to regulate plant growth and stress responses. Thus, glutamine is an important nutrient and a functional amino acid in plants.

## Availability of supporting data

The data set supporting the results of this article is available in the NCBI GEO repository [http://www.ncbi.nlm.nih.gov/geo/query/acc.cgi?acc=GSE56770].

## Additional file

Additional file 1: Table S1. Primers used for RT-PCR analysis of glutamine-responsive genes. **Table S2.** Primers used for quantitative RT-PCR analysis of glutamine-responsive transcription factor genes. **Figure S1.** Effects of alanine and glycine on the growth of rice seedlings. **Figure S2.** Amino acid sequence alignment of Arabidopsis LBD37 and its rice orthologs Os03g0445700 and Os07g0589000. (PDF 2445 kb)

## Abbreviations

GS: Glutamine synthetase
GOGAT: Glutamine-oxoglutarate aminotransferase
NAGK: N-acetyl-glutamate kinase
mTORC1: Mammalian target of rapamycin complex 1
RT-PCR: Reverse transcription-polymerase chain reaction
RR: Response regulator
PUP: Purine transporters
ROS: Reactive oxygen species
