# Supplementary material for: Glutamine rapidly induces the expression of key transcription factor genes involved in nitrogen and stress responses in rice roots
Source: BMC Genomics. 2015 Sep 25;16:731. doi: 10.1186/s12864-015-1892-7 (PMC4582844; doi:10.1186/s12864-015-1892-7)
Supplement: Additional file 1: Table S1. — Primers used for RT-PCR analysis of glutamine-responsive genes. Table S2. Primers used for quantitative RT-PCR analysis of glutamine-responsive transcription factor genes. Figure S1. Effects of alanine and glycine on the growth of rice seedlings. Figure S2. Amino acid sequence alignment of Arabidopsis LBD37 and its rice orthologs Os03g0445700 and Os07g0589000. (PDF 2445 kb) [file 12864_2015_1892_MOESM1_ESM.pdf]

Table S1 List of primers used for RT-PCR analysis

Gene	Primer sequence 5'→3'
ZOS5-02 *Os05g0114400*	ATCATGAAGAGGTTTGCATT/ CCGGCAAAGAGAGCTAGTA
DREB1A *Os09g0522200*	GAGCATGCTGCCTCAACTTC/TCCATCACATTACCGAAAGT
NAC5 *Os11g0184900*	TGATTGGGTGTTGTGCCGAA/TTTGCAGAGGCGTCACGTTC
GAD3 *Os03g0236200*	GAAGACGGGGAGGTTCGACA/GTGGCGCTGTCGCTAATTGA
GDU-like *Os06g0633100*	CGAGAGAGGCAAACACAC/AATCTCACGAACAAAATTGG
BBTI13 *Os03g0823400*	AACGAAAGCGAGAGATGAG/GTGGGTCGGGTCAGATGG
EF-hand *Os09g0482800*	AGCCAAGAATTCGATCGGGC/AGGCAGAATCTCTGACGCCT
IRO2 *Os01g0952900*	TCTTCCATCTGCAAGCTCGAG/TACAACTAGCGTAGTGTGGTGG
WRKY69 *Os08g0386200*	CCTCTTCGACGTCGTCTACC/CTCTGCTATGGCTTTTCTTC
MAPKKK57 *Os05g0545300*	CGAAGCCCAAGTGGGTCTCT/TTTCATCCCAAGCACTCGCG
RR6 *Os04g0673300*	TGGATTGGATTATTGGGTTGGA/CGAGCGAGCGATCATCTGAT
Unknown *Os08g0172900*	ATGGCCATGACCTTGCCG/TCAATGGATCTTTGGGCC
LBD37-like *Os03g0445700*	CATCACCATGAGCTGCAACGG/CCGGCCTCAGACAAAAAGGTT
UCC1-like *Os08g0138100*	ATGGCAGCTGCCAACAGA/ TTAGAAGGACATGAGGCCAG
AP2/ERF106 *Os07g0410300*	CAGCTCACAGAGTCACACAC/TTCGTTCGCATCATCATC

DUF581 *Os02g0687200*	TCAGCTCGAGTTGTAAGTTG/ATGCTTTACTTGCGCCTAT
MYB-like *Os07g0119300*	TCTTCAGAAGTACCGCCTGC/GCCGCAATTAACCTAACCCAC
RLK-like *Os03g0564600*	TCTTGAGGCGGCAGCATTCC/GCTTCCACCCAACACTTTGC
PFK04 *Os05g0194900*	TGTGCGCCAAGATCAAGGATCAC/AGCTACTTCTTCAAGCTTCAGGC
DUF966 *Os01g0975000*	CCGTGCTACTGCTTGGCATA/CGATGGATGAGTTGCCTGACA
GH17 *Os02g0532900*	GGACGTACATCGGGAACCTC/CAGTGGCTGATACTGTCACGA
EDGP-like *Os05g0402900*	GTCCACCAAGGGAGACAAATC/CATCTAGCCGAGGCGAAAGT
MAPKKK63 *Os01g0699100*	CACACTGGACGCCGCATTCT/GAAAGCGTCCGCTTCATTGCTT
LBD37-like *Os07g0589000*	GTGCTCCACGTTCTCGAC/CAGCAAAGAAAGCATATGAG
CIPK14 *Os12g0113500*	CAGGTGTTGAAGGTCGGGC/AGTTTGCGTGAAAACCAACCGG
HMA protein *Os02g0585100*	ATGGATGTCGAGCCTTCCAG/CTACATGATGTAGCCGTAAG
UCC1-like *Os08g0138200*	ATGGCAGCATCAGCCAGA/CTAATAAAAGACCATGAGACCAGC
bHLH *Os04g0301500*	AACTCGAAGCAGAGCAAA/CATATATCTATCCAGCTCATAA
KCS11 *Os02g0205500*	GTACGAGCTGGCGTACAG/CTAACAAAAGTGCCAATTCC
PUP3-like *Os03g0187800*	GATTCGCCTCCTACCTCTAC/GTATACGACCCACCCAAGTA
OsWAK21 *Os02g0807900*	ACGATGAAAGAGGTAGCAGA/AAAAGATTAACGCAAACAACA
ABC transporter	CTATCCAAAGTGGGCATTAG/TCAAATGTGAAAAGTTGGTG

Os04g0194500	
DUF668 *Os01g0845000*	CGAGATGTACGAGATGCTG/ATCTTTGGGCAAAAGTTTC
GDU-like *Os08g0446800*	ATGAGGCCAGGAGCAGGG/TCACACCTCAGGGATGTGG
Unknown *Os03g0124800*	GAGACAGCCCAAAAGCTGAG/TTACACTTGAAAACCAGCTG
EF1α	CGTGAGAGAGGTATCACCAT/GGAATCTTGTCAGGGTTGTA

Table S2 List of primers used for quantitative RT-PCR analysis

Gene	Primer sequence 5'→3'
DREB1A *Os09g0522200*	TTCAGGAATCAGGAGCAAGCA/CCGCACATCTTCGGATTTGT
NAC5 *Os11g0184900*	GAGCGAGGTTAAAAATAGTGGAGAA/CCATCCGGCAGAAACAAAAA
ZOS5-02 *Os05g0114400*	AGTTCGCCATTGGCCAGGCTCTC/GTGGATGGTTCAGGTCGAGCCACAG
IRO2 *Os01g0952900*	TGTACAGTCAATTGGCGAATGG/TGTGCCGTGTTTTCTCGCT
WRKY69 *Os08g0386200*	TCCTTTTGTTTCTCCCAACTCTCT/CCTCTCTTTTGCTTGACAGAAACC
LBD37-like *Os03g0445700*	TCGTGCGACCTAGGCCTGTG/CCGGCCTCAGACAAAAAGGTT
AP2/ERF106 *Os07g0410300*	ACTAGCCATTGATGATGATG/AATCCACTACCAACTGATCG
MYB-like *Os07g0119300*	AAAGCAGCTCCCAGTCTGGAT/CCATGCCTGAGCCTGAGAAA
LBD37-like *Os07g0589000*	CCTGCTCAATTCTCATATGCTTTC/GGCGAGAGGCTACCAAAGAGT
bHLH *Os04g0301500*	GTCATTTTTGCGACACCCCA/CCTCGCAAGAAAACAACCACC
UBC3 *Os02g0634800*	AAGGCATGGCTGATTCATGT/CAGATGTTCACATGGTGACAGTA

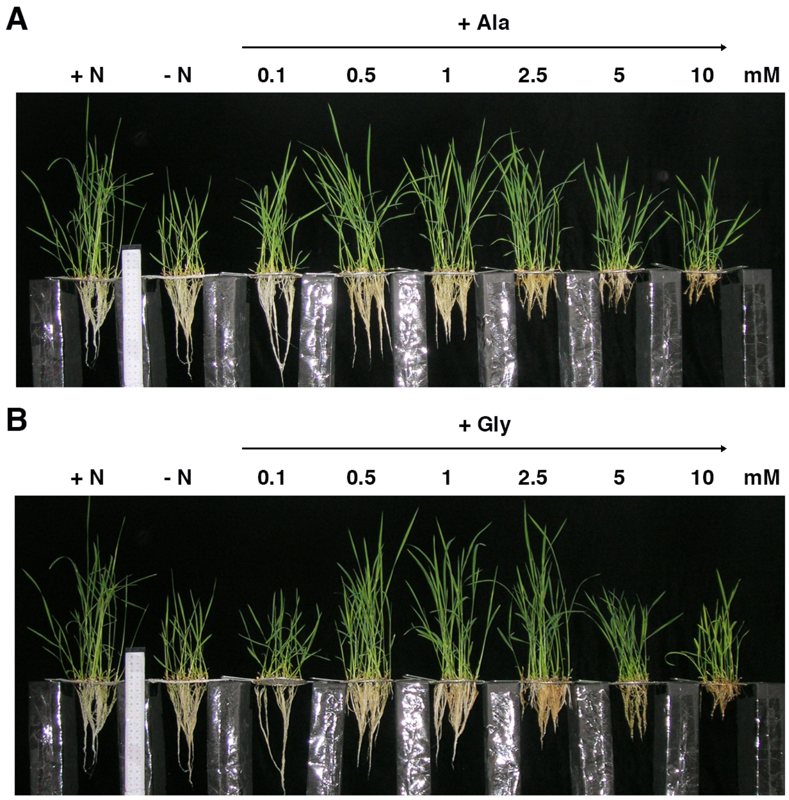

Figure S1 Effects of amino acids on the growth of rice seedlings. Supplementation with alanine and glycine at optimal concentrations can promote rice growth. Plants shown are 17 days old rice seedlings grown in hydroponic solutions with 1.43 mM NH_4NO_3 (+ N), without nitrogen (- N) or containing 0.1 – 10 mM alanine (A) or glycine (B) as the sole nitrogen source.

```
AtLBD37           1 MSCNGCRVLRKGCSENCILRPCIQWIETADAQGHATVFVAKFFGRAGLMSFISAVPDSQR
Os03g0445700      1 MSCNGCRVLRKGCSDGCVLRPCLQWIDAADAQGHATVFVAKFFGRAGLLSFISAVPEAQR
OS07G0589000      1 MSCNGCRVLRKGCSEGCVLRPCLQWIDGAEAQGHATVFVAKFFGRAGLMSFLTAVPEPQR

AtLBD37          61 PALFQSLLYEACGRTVNPVNGAIGMLWTGNWNICQAAVETVLRGGSLRPIPELLTHGGGF
Os03g0445700     61 PALFQSLLYEAAGRTINPVHGAVGLLWTGNWPLCQAAVETVLRGGAIGPLPELGGACGGA
OS07G0589000     61 AAVFQSLLYEAAGRTINPVGGAVGLLSGGSWHLCQAAVDTVLRGGGIQPLPDQVDAAAAG

AtLBD37         121 AG--FPSPTSEEASEICTEMLNLQQNDSTDRNIYHHSRFSSSR-S---RSTMDSSSPTKR
Os03g0445700    121 GGDLYGAA---KRNGGW-------------------STFSTAKRVRKA------------
OS07G0589000    121 GRDVFASTA-RRAMGGC-------------------STFSTAKRSTTTTTTKNPGTPHDA

AtLBD37         175 KRLSSEDQPSSELDLSLIPNFPIKQATPSSTRRRSVTPSMNSEDSGTTTTTTAFCDKGDV
Os03g0445700    147 ---EVPEAPSCDLGLCLSPGSPPAVGERKPAL-RPGTPSMSSDES----GTT--------
OS07G0589000    161 AAAAPQPEPSCDLGLWLSPGSPPAPGDRRSGGRRADTPSMNSEGSVTTCGVV--------

AtLBD37         235 YGNGGGETTKLLNLFV
Os03g0445700    191 --TGGERDPVLLNLFV
OS07G0589000    213 --GDGEREPELLNLFV
```

Figure S2 Amino acid sequence alignment of Arabidopsis LBD37 and its rice orthologs Os03g0445700 and Os07g0589000. The sequences were aligned with Clustal Omega (http://www.ebi.ac.uk/Tools/msa/clustalo/) and shaded with BoxShade (http://www.ch.embnet.org/software/BOX_form.html). Identical and conserved amino acids are shaded in black and grey, respectively. AtLBD37, NP_201543; Os03g0445700, NP_001050468; Os07g0589000, NP_001060148.
